# Supplementary material for: Activated-memory T cells influence naïve T cell fate: a noncytotoxic function of human CD8 T cells
Source: Commun Biol. 2022 Jun 29;5:634. doi: 10.1038/s42003-022-03596-2 (PMC9243096; doi:10.1038/s42003-022-03596-2)
Supplement: Supplementary file 2 — Supplementary Information [file 42003_2022_3596_MOESM2_ESM.pdf]

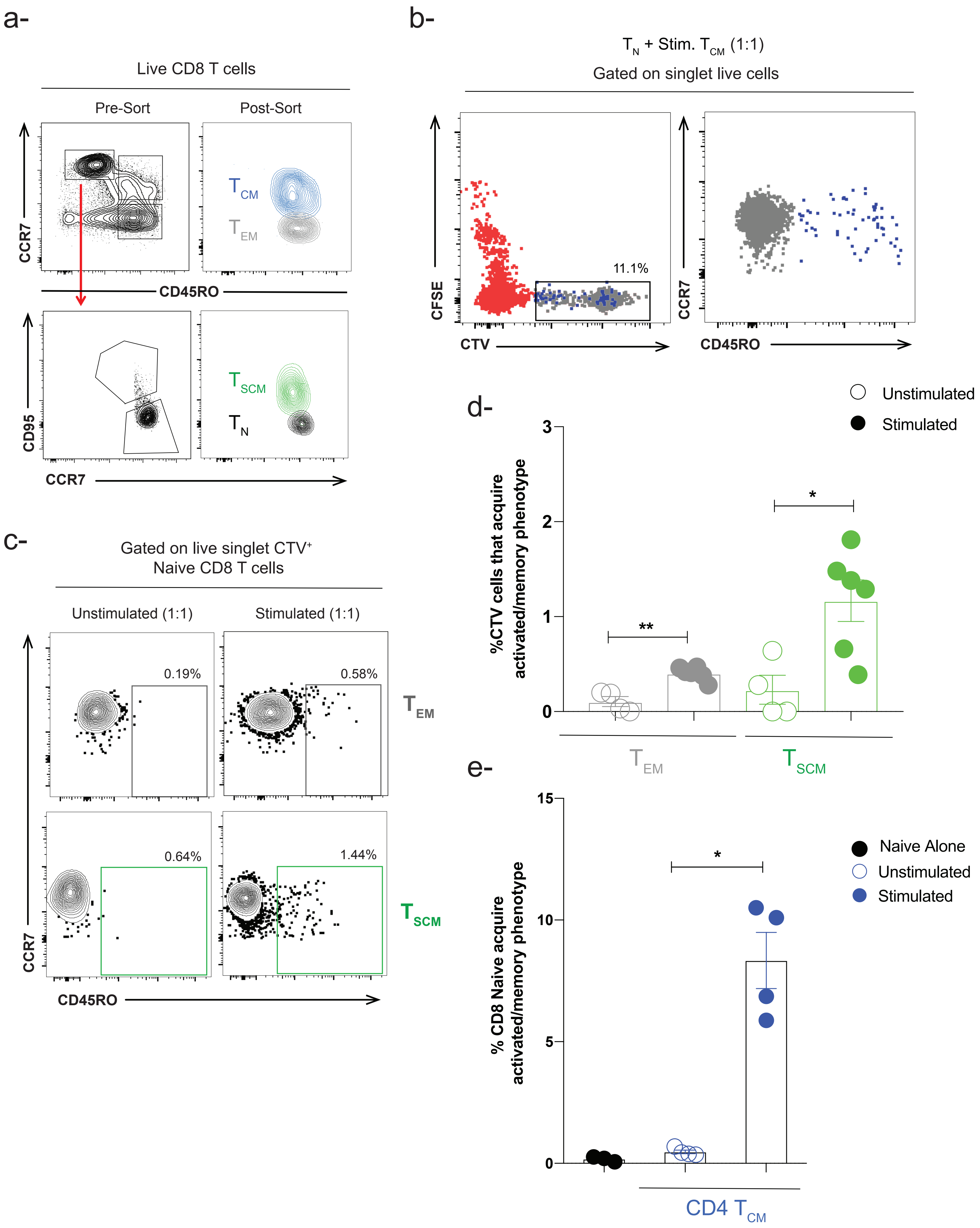

a-
Live CD8 T cells
Pre-Sort
Post-Sort
CCR7
CD45RO
TCM
TEM
CD95
CCR7
TSCM
TN
b-
TN + Stim. TCM (1:1)
Gated on singlet live cells
CFSE
CTV
11.1%
CCR7
CD45RO
c-
Gated on live singlet CTV+
Naive CD8 T cells
Unstimulated (1:1)
Stimulated (1:1)
0.19%
0.58%
0.64%
1.44%
TEM
TSCM
CCR7
CD45RO
d-
Unstimulated
Stimulated
%CTV cells that acquire
activated/memory phenotype
**
*
TEM
TSCM
e-
% CD8 Naive acquire
activated/memory phenotype
Naive Alone
Unstimulated
Stimulated
*
CD4 TCM

Supplementary Figure 1: a Representative contour flow plots showing flow cytometry-based strategy for isolation of naïve and memory CD8 T cell subsets from lymphocytes of healthy adults. The cell subsets were identified based on differential expression of three cell surface markers as follows: Naïve: CCR7^{+}, CD45RO^{-}, and CD95^{-}; T_{EM}: CCR7^{-}and CD45RO^{+}; T_{CM}: CCR7^{+}, and CD45RO^{+}; and T_{SCM}: CCR7^{+}, CD45RO^{-},and CD95^{+}. **b** Representative flow contour plot showing the distribution of T_N CD8 T cells with acquired activated/memory phenotype within the CTV$^{+/Int.}$ population. **c** Representative flow contour plots and **d** Bar-graph showing percent of CTV-labeled naïve CD8 T cells that acquire activated/memory phenotype based on CCR7 and CD45RO cell surface expression (n=4-6) in the presence of unstimulated or stimulated T_{EM} (grey box), or T_{SCM} (green box) CD8 T cells (memory: naïve - 1:1 ratio). **e** Bar-graph showing percent of naïve CD8 T cells that acquire activated/memory phenotype based on CCR7 and CD45RO cell surface expression in the presence (n=4) of unstimulated or stimulated T_{CM} CD4 T cells (blue box), at memory: naïve (1:1 ratio). Closed circles depict stimulation conditions while open circles depict unstimulated conditions *$P<0.05$ **$P<0.01$ Unpaired non-parametric Mann-Whitney test was used. Data were presented as means +/- SEM.

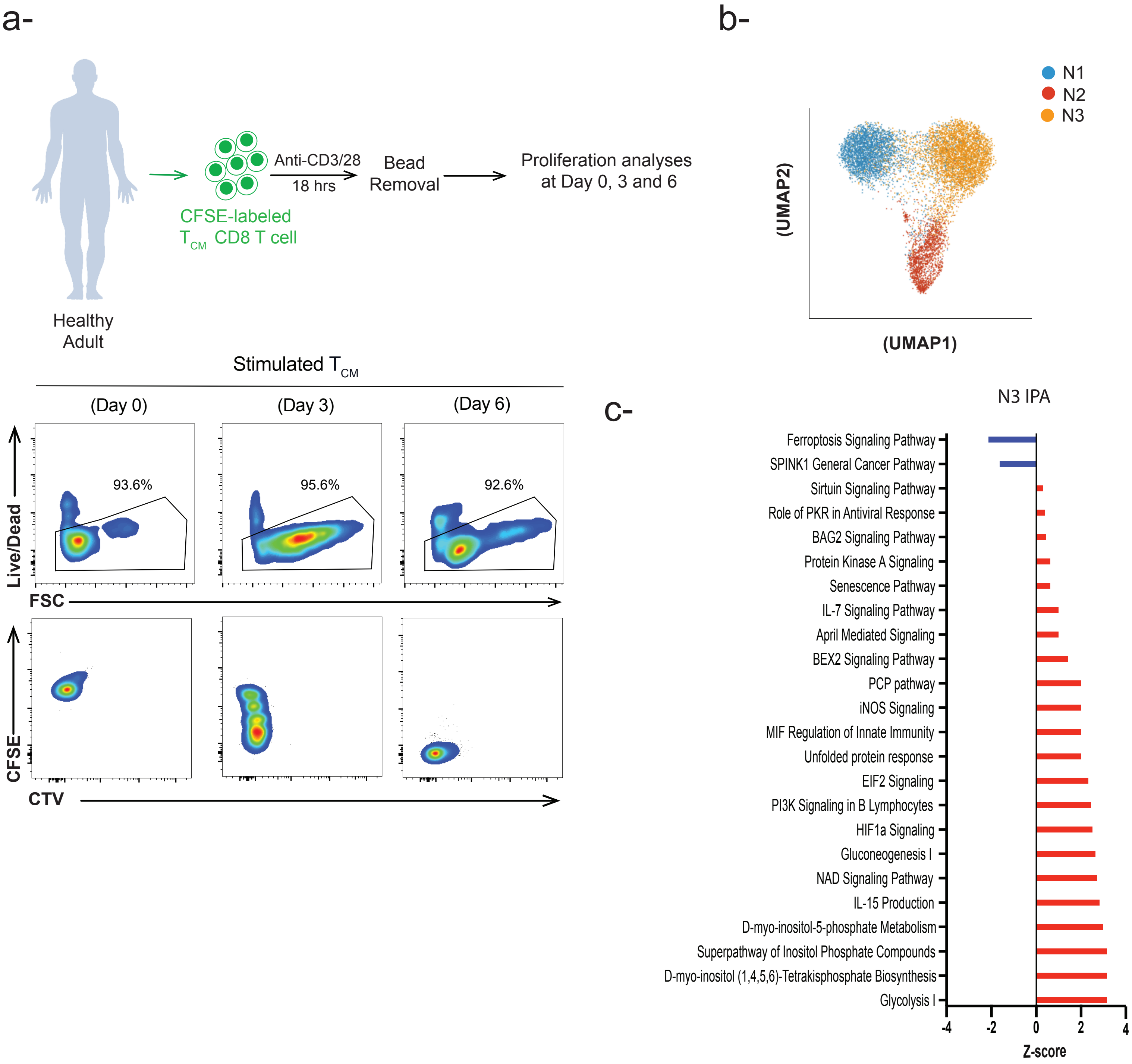

Supplementary Figure 2: a *In vitro* schema and FACS-plots showing stimulation of sorted CFSE-labeled human T_{CM} CD8 T cell subset with anti-CD3/CD28 magnetic beads (1:1 ratio). After overnight stimulation, the beads were removed using a plate magnet followed by culturing activated T_{CM} CD8 T cell without further manipulation. Proliferation capacity was measured at Day 0, 3, 6 incubations. **b** UMAP analyses focusing on the top 2000 genes showing Blue neighborhood-N1, Red neighborhood-N2, and Orange neighborhood-N3. **c** Ingenuity pathway analyses (IPA) of the unique DEG list in N3 (Orange neighborhood) showing top pathways based on Z-score.

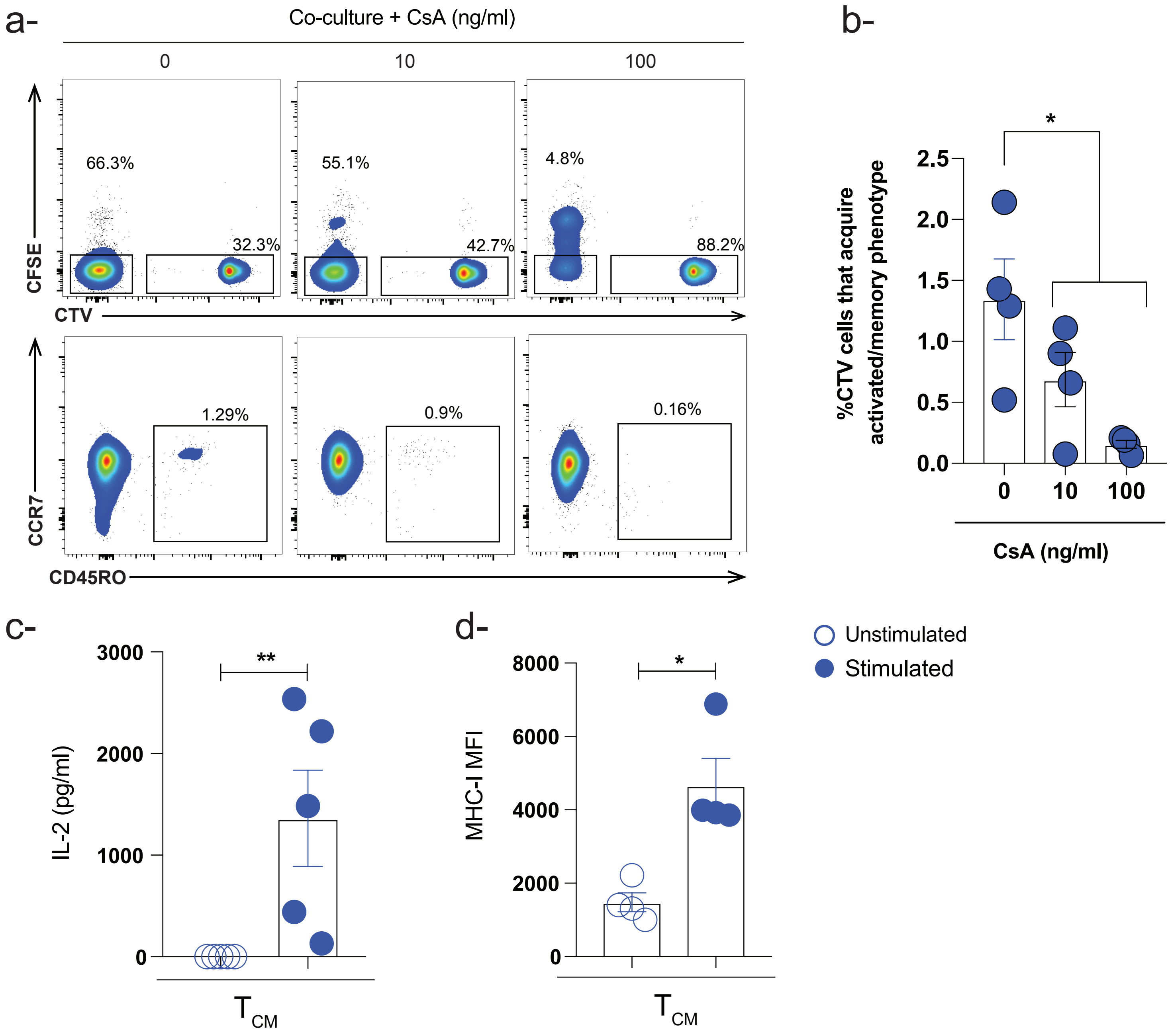

e-

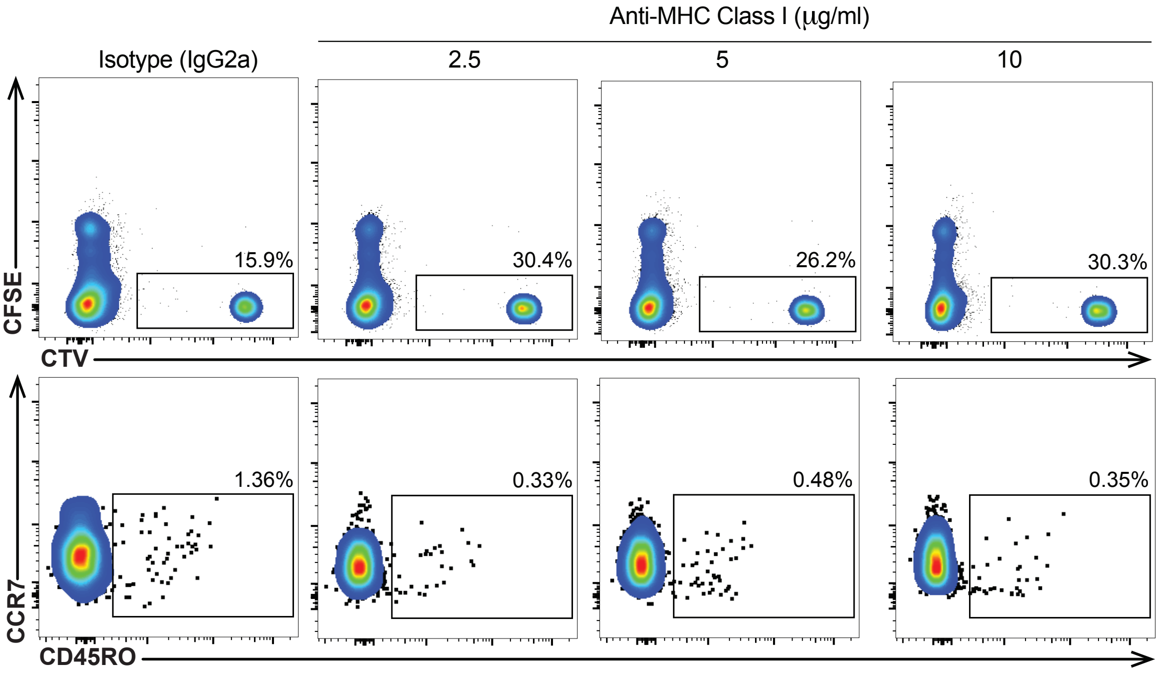

Supplementary Figure 3: a Representative flow plots and **b** Bar-graph showing the effect of Cyclosporin A (CsA) on proliferation of T_{CM} CD8 T cells as well as naïve cells with activated/memory phenotype (n=4). **c** Bar-graph showing IL-2 concentrations in the supernatant of stimulated T_{CM} CD8 cells (7 days of following 18 hrs of beads stimulation) compared to unstimulated controls (n=5). **d** Bar-graph showing MFI of MHC-I in unstimulated and stimulated T_{CM} CD8 T cells following overnight beads removal (n=4). **e** Representative flow plots showing the effect of blocking MHC-I on acquisition of activated/memory phenotype by naïve CD8 T cells in the presence of activated T_{CM} CD8 T cells. **P<0.05 **P<0.01* Unpaired non-parametric Mann-Whitney test was used (Panels c-d). Ordinary one-way ANOVA Bonferroni multiple comparison test was used **P<0.05*. Data were presented as means +/-SEM (Panel b).

		N2 vs N1		N3 Vs N1		N3 vs N2	
	Gene ID	P-value	Fold change	P-value	Fold change	P-value	Fold change
**Effector cytokines, molecules, & TF**	***GZMA***	0.00	17.98	0.70	1.15	0.00	-15.58
	GZMB	0.00	13.81	0.61	1.00	0.00	-13.78
	GZMK	0.00	13.02	0.55	1.33	0.00	-9.82
	PRF1	0.00	10.28	0.96	1.05	0.00	-9.82
	IFNG	0.00	6.33	0.74	1.13	0.00	-5.60
	IL4	0.00	3.03	0.56	-1.22	0.00	-3.70
	TBX21	0.00	7.48	0.69	1.19	0.00	-6.29
	ID2	0.00	4.42	0.81	1.14	0.00	-3.88
	ZEB2	0.00	6.56	0.58	1.28	0.00	-5.12
	RUNX3	0.00	4.06	0.00	2.01	0.00	-2.02
	GATA3	0.00	3.12	0.56	1.05	0.00	-2.98
	EOMES	0.00	2.65	0.24	-1.30	0.00	-3.44
	HOPX	0.00	9.19	0.89	1.13	0.00	-8.14
	PRDM1	0.00	5.69	0.46	1.29	0.00	-4.42
	IRF4	0.00	4.32	0.73	1.01	0.00	-4.29
	TCF7	0.00	-12.95	0.00	-1.81	0.00	7.17
	LEF1	0.00	-5.42	0.00	-1.07	0.00	5.08
	FOXP1	0.00	-2.07	0.00	1.18	0.00	2.45
**Glycolysis**	***HK2***	0.00	3.21	0.00	7.48	0.19	2.33
	TPI1	0.00	4.30	0.00	3.33	0.00	-1.29
	LDHA	0.00	4.15	0.00	4.55	0.01	1.10
	ALDOA	0.00	2.46	0.00	4.11	0.00	1.67
	SLC2A3	0.00	8.82	0.00	3.79	0.00	-2.33
**Miscellaneous**	***CD69***	5.59717E-319	4.94	0.00	2.41	0.00	-2.05
	CD95	0.00	6.03	0.34	1.21	0.00	-4.98
	HNRNPLL	0.00	3.01	0.04	-1.15	0.00	-3.47
	LTB	0.00	2.27	0.00	-1.22	0.00	-2.78
	SLAMF7	0.00	10.23	0.32	1.46	0.00	-6.99
	PASK	0.00	-5.74	0.00	-4.77	0.00	1.20
	LRRN3	0.00	-4.27	0.00	-5.64	0.00	-1.32
	IL6R	0.00	-5.68	0.00	-1.55	0.00	3.66
	IL6ST	0.00	-2.57	0.23	1.14	0.00	2.94
	IL7R	0.00	-5.56	0.00	-1.68	0.00	3.31
	NOSIP	0.00	-1.06	0.00	1.37	0.00	1.45
	NUR77	0.00	3.12	0.09	1.58	0.00	-1.98

Supplementary Table 1: Table showing fold change and P values for the gene list described in Figure 6d heat maps.
