# Supplementary material for: Activated-memory T cells influence naïve T cell fate: a noncytotoxic function of human CD8 T cells
Source: Commun Biol. 2022 Jun 29;5:634. doi: 10.1038/s42003-022-03596-2 (PMC9243096; doi:10.1038/s42003-022-03596-2)
Supplement: Supplementary file 5 — Reporting Summary [file 42003_2022_3596_MOESM5_ESM.pdf]

nature portfolio

Corresponding author(s): Hossam A. Abdelsamed

Last updated by author(s): Oct 10, 2021

Reporting Summary

Nature Portfolio wishes to improve the reproducibility of the work that we publish. This form provides structure for consistency and transparency in reporting. For further information on Nature Portfolio policies, see our Editorial Policies and the Editorial Policy Checklist.

Statistics

For all statistical analyses, confirm that the following items are present in the figure legend, table legend, main text, or Methods section.

n/a	Confirmed	
☐	☒	The exact sample size (n) for each experimental group/condition, given as a discrete number and unit of measurement
☐	☒	A statement on whether measurements were taken from distinct samples or whether the same sample was measured repeatedly
☐	☒	The statistical test(s) used AND whether they are one- or two-sided<br>*Only common tests should be described solely by name; describe more complex techniques in the Methods section.*
☒	☐	A description of all covariates tested
☒	☐	A description of any assumptions or corrections, such as tests of normality and adjustment for multiple comparisons
☐	☒	A full description of the statistical parameters including central tendency (e.g. means) or other basic estimates (e.g. regression coefficient) AND variation (e.g. standard deviation) or associated estimates of uncertainty (e.g. confidence intervals)
☒	☐	For null hypothesis testing, the test statistic (e.g. F, t, r) with confidence intervals, effect sizes, degrees of freedom and P value noted<br>*Give P values as exact values whenever suitable.*
☒	☐	For Bayesian analysis, information on the choice of priors and Markov chain Monte Carlo settings
☒	☐	For hierarchical and complex designs, identification of the appropriate level for tests and full reporting of outcomes
☒	☐	Estimates of effect sizes (e.g. Cohen's d, Pearson's r), indicating how they were calculated

Our web collection on statistics for biologists contains articles on many of the points above.

Software and code

Policy information about availability of computer code

Data collection	Single cell raw matrix files were obtained using the Cell Ranger's pipeline (10X genomics) with alignment to the human reference hg38.<br>All flow cytometery data was acquired using FACSDiva software.
Data analysis	scRNA-Seq raw matrix data was preprocessed and analyzed using Partek Flow (Version 10.0.21.0801).<br>Statistical analyses were performed using GraphPad Prism (Version 8.3).<br>Flow analyses were performed using FlowJo (Version 10.5.3).<br>Image stream analyses were performed using Amnis IDEAS software (Luminex).

For manuscripts utilizing custom algorithms or software that are central to the research but not yet described in published literature, software must be made available to editors and reviewers. We strongly encourage code deposition in a community repository (e.g. GitHub). See the Nature Portfolio guidelines for submitting code & software for further information.

Data

Policy information about availability of data

All manuscripts must include a data availability statement. This statement should provide the following information, where applicable:

- Accession codes, unique identifiers, or web links for publicly available datasets
- A description of any restrictions on data availability
- For clinical datasets or third party data, please ensure that the statement adheres to our policy

Authors confirm that all relevant data were included in the article and/or its supplementary information files as well as links to publicly available data sets before publishing. GEO Accession number GSE202548.

Field-specific reporting

Please select the one below that is the best fit for your research. If you are not sure, read the appropriate sections before making your selection.

☒ Life sciences ☐ Behavioural & social sciences ☐ Ecological, evolutionary & environmental sciences

For a reference copy of the document with all sections, see nature.com/documents/nr-reporting-summary-flat.pdf

Life sciences study design

All studies must disclose on these points even when the disclosure is negative.

Sample size	In our study, since we are working with a small cohort of healthy adult subjects, we expect that our sample size will be less than n=30. Consequently, our sample size will follow t-student distribution statistical analyses.
Data exclusions	Sorting experiments with efficiency less than 95% purity were excluded. In some incidences, the sorted sample undergo another round of sorting to reach purity above 95%. However, we lose ~50% of the sample cell number but did not observe any effect on the sample viability or change in the phenotype.
Replication	To verify reproducibility, same cell staining protocol (Antibody conc., incubation time, spinning speed and time) along with same clones of flurochrome-conjugated antibodies were used in each experiment. Additionally, flow cytometry data were acquired on the same flow cytometer (BD LSRFortessa) using same experimental template and voltage settings. Color compensation is checked and confirmed in each experiment. Finally, independent reproducibility has been achieved by performing the experiments through more than one individual in the lab.
Randomization	Samples from healthy adult human subjects were allocated randomly. Only samples with low cell viability to start with were excluded from the experiments.
Blinding	Blinding will not be relevant in our study design since we are not reporting or working with a clinical trial where randomized single or double blinded approaches is required. To limit biasness in our experimental design, we set our gating strategies based on the controls i.e., naive alone or unstimulated memory T cell.

Reporting for specific materials, systems and methods

We require information from authors about some types of materials, experimental systems and methods used in many studies. Here, indicate whether each material, system or method listed is relevant to your study. If you are not sure if a list item applies to your research, read the appropriate section before selecting a response.

Materials & experimental systems

n/a	Involved in the study	
☐	☒	Antibodies
☒	☐	Eukaryotic cell lines
☒	☐	Palaeontology and archaeology
☒	☐	Animals and other organisms
☐	☒	Human research participants
☒	☐	Clinical data
☒	☐	Dual use research of concern

Methods

n/a	Involved in the study	
☐	☐	ChIP-seq
☐	☒	Flow cytometry
☐	☐	MRI-based neuroimaging

Antibodies

Antibodies used	Live-Dead exclusion dye (Ghost dye Violet 510, TONBO), CD8-APCCy7 (Biolegend-Clone:SK1), CCR7-PE (Biolegend-Clone:G043H7), CD45RO-APC (Biolegend-Clone:UCHL1), and CD95-PECy7 (Biolegend-Clone:DX2)

Validation	Staining and validation were done per manufacturer's recommendations.

Human research participants

Policy information about studies involving human research participants

Population characteristics	All participants were adult male or female healthy subjects with age less than 65.
Recruitment	All participants were recruited as part of our approved IRB protocol at the Starzl Transplantation Institute (IRB#00608014)
Ethics oversight	University of Pittsburgh-School of Medicine

Note that full information on the approval of the study protocol must also be provided in the manuscript.

ChIP-seq

Data deposition

☐ Confirm that both raw and final processed data have been deposited in a public database such as GEO.

☐ Confirm that you have deposited or provided access to graph files (e.g. BED files) for the called peaks.

Data access links<br>*May remain private before publication.*	*For "Initial submission" or "Revised version" documents, provide reviewer access links. For your "Final submission" document, provide a link to the deposited data.*
Files in database submission	*Provide a list of all files available in the database submission.*
Genome browser session<br>(e.g. UCSC)	*Provide a link to an anonymized genome browser session for "Initial submission" and "Revised version" documents only, to enable peer review. Write "no longer applicable" for "Final submission" documents.*

Methodology

Replicates	*Describe the experimental replicates, specifying number, type and replicate agreement.*
Sequencing depth	*Describe the sequencing depth for each experiment, providing the total number of reads, uniquely mapped reads, length of reads and whether they were paired- or single-end.*
Antibodies	*Describe the antibodies used for the ChIP-seq experiments; as applicable, provide supplier name, catalog number, clone name, and lot number.*
Peak calling parameters	*Specify the command line program and parameters used for read mapping and peak calling, including the ChIP, control and index files used.*
Data quality	*Describe the methods used to ensure data quality in full detail, including how many peaks are at FDR 5% and above 5-fold enrichment.*
Software	*Describe the software used to collect and analyze the ChIP-seq data. For custom code that has been deposited into a community repository, provide accession details.*

Flow Cytometry

Plots

Confirm that:

☒ The axis labels state the marker and fluorochrome used (e.g. CD4-FITC).

☒ The axis scales are clearly visible. Include numbers along axes only for bottom left plot of group (a 'group' is an analysis of identical markers).

☒ All plots are contour plots with outliers or pseudocolor plots.

☒ A numerical value for number of cells or percentage (with statistics) is provided.

Methodology

Sample preparation	Sample preparation was performed as detailed in the methods section.
Instrument	BD LSRFortessa was used for collecting flow cytometry data. BD FACSAria II was used for sorting the relevant cell populations.
Software	BD FACSDiva was used for data acquisition. FlowJo was used for data analyses.
Cell population abundance	Naive and memory CD8 T cell subsets were abundant except for stem cell memory (Tscm) albeit in some cases Tscm cell

Cell population abundance	number was just enough for our downstream analyses. Sorting experiments that result in 95% purity or more were used.
Gating strategy	Gates in FSC/SSC plots were drawn to in a way to exclude cell debris/dead cells followed by singlets gate (FSC-A vs SSC-W). Live/dead gating was used to further exclude apoptotic/necrotic cells. we set our gating strategies based on the controls i.e., naive alone or unstimulated memory T cell. The gating strategy we used was published before by other labs including corresponding author publication. The detailed gating strategy is described in the methods section along with cited references.

☒ Tick this box to confirm that a figure exemplifying the gating strategy is provided in the Supplementary Information.

Magnetic resonance imaging

Experimental design

Design type	*Indicate task or resting state; event-related or block design.*
Design specifications	*Specify the number of blocks, trials or experimental units per session and/or subject, and specify the length of each trial or block (if trials are blocked) and interval between trials.*
Behavioral performance measures	*State number and/or type of variables recorded (e.g. correct button press, response time) and what statistics were used to establish that the subjects were performing the task as expected (e.g. mean, range, and/or standard deviation across subjects).*

Acquisition

Imaging type(s)	*Specify: functional, structural, diffusion, perfusion.*
Field strength	*Specify in Tesla*
Sequence & imaging parameters	*Specify the pulse sequence type (gradient echo, spin echo, etc.), imaging type (EPI, spiral, etc.), field of view, matrix size, slice thickness, orientation and TE/TR/flip angle.*
Area of acquisition	*State whether a whole brain scan was used OR define the area of acquisition, describing how the region was determined.*

Diffusion MRI ☐ Used ☐ Not used

Preprocessing

Preprocessing software	*Provide detail on software version and revision number and on specific parameters (model/functions, brain extraction, segmentation, smoothing kernel size, etc.).*
Normalization	*If data were normalized/standardized, describe the approach(es): specify linear or non-linear and define image types used for transformation OR indicate that data were not normalized and explain rationale for lack of normalization.*
Normalization template	*Describe the template used for normalization/transformation, specifying subject space or group standardized space (e.g. original Talairach, MNI305, ICBM152) OR indicate that the data were not normalized.*
Noise and artifact removal	*Describe your procedure(s) for artifact and structured noise removal, specifying motion parameters, tissue signals and physiological signals (heart rate, respiration).*
Volume censoring	*Define your software and/or method and criteria for volume censoring, and state the extent of such censoring.*

Statistical modeling & inference

Model type and settings	*Specify type (mass univariate, multivariate, RSA, predictive, etc.) and describe essential details of the model at the first and second levels (e.g. fixed, random or mixed effects; drift or auto-correlation).*
Effect(s) tested	*Define precise effect in terms of the task or stimulus conditions instead of psychological concepts and indicate whether ANOVA or factorial designs were used.*

Specify type of analysis: ☐ Whole brain ☐ ROI-based ☐ Both

Statistic type for inference (See Eklund et al. 2016)	*Specify voxel-wise or cluster-wise and report all relevant parameters for cluster-wise methods.*
Correction	*Describe the type of correction and how it is obtained for multiple comparisons (e.g. FWE, FDR, permutation or Monte Carlo).*

Models & analysis

n/a	Involved in the study
☐	☐ Functional and/or effective connectivity
☐	☐ Graph analysis
☐	☐ Multivariate modeling or predictive analysis

Functional and/or effective connectivity	*Report the measures of dependence used and the model details (e.g. Pearson correlation, partial correlation, mutual information).*
Graph analysis	*Report the dependent variable and connectivity measure, specifying weighted graph or binarized graph, subject- or group-level, and the global and/or node summaries used (e.g. clustering coefficient, efficiency, etc.).*
Multivariate modeling and predictive analysis	*Specify independent variables, features extraction and dimension reduction, model, training and evaluation metrics.*
